# Comprehensive analysis of PSME3: from pan-cancer analysis to experimental validation

**DOI:** 10.3389/fimmu.2024.1295693

**Published:** 2024-01-19

**Authors:** Chengyuan Dong, Yadong Guo, Yanrong Yang, Xin Ge

**Affiliations:** ^1^ Department of Clinical Medicine, Shanghai Tenth People’s Hospital, School of Medicine, Tongji University, Shanghai, China; ^2^ School of Medicine, Anhui University of Science and Technology, Huainan, China; ^3^ Department of Urology, Shanghai Tenth People’s Hospital, School of Medicine, Tongji University, Shanghai, China; ^4^ Tongji University Cancer Center, School of Medicine, Tongji University, Shanghai, China

**Keywords:** immunotherapy, PSME3, tumor markers, CD276, LIHC

## Abstract

PSME3 plays a significant role in tumor progression. However, the prognostic value of PSME3 in pan-cancer and its involvement in tumor immunity remain unclear. We conducted a comprehensive study utilizing extensive RNA sequencing data from the TCGA (The Cancer Genome Atlas) and GTEx (Genotype-Tissue Expression) databases. Our research revealed abnormal expression levels of PSME3 in various cancer types and unveiled a correlation between high PSME3 expression and adverse clinical outcomes, especially in cancers like liver cancer (LIHC) and lung adenocarcinoma (LUAD). Functional enrichment analysis highlighted multiple biological functions of PSME3, including its involvement in protein degradation, immune responses, and stem cell regulation. Moreover, PSME3 showed associations with immune infiltration and immune cells in the tumor microenvironment, indicating its potential role in shaping the cancer immune landscape. The study also unveiled connections between PSME3 and immune checkpoint expression, with experimental validation demonstrating that PSME3 positively regulates CD276. This suggests that PSME3 could be a potential therapeutic target in immunotherapy. Additionally, we predicted sensitive drugs targeting PSME3. Finally, we confirmed in both single-factor Cox and multiple-factor Cox regression analyses that PSME3 is an independent prognostic factor. We also conducted preliminary validations of the impact of PSME3 on cell proliferation and wound healing in liver cancer. In summary, our study reveals the multifaceted role of PSME3 in cancer biology, immune regulation, and clinical outcomes, providing crucial insights for personalized cancer treatment strategies and the development of immunotherapy.

## 1 Introduction

Cancer, a leading cause of mortality in the world, is a persistent public health issue (1, 2). Immunotherapy has emerged as a promising approach for treating various cancer types, such as melanoma, lung cancer, and lymphoma (3–5). However, not all patients respond equally to immunotherapy (6), and there is a need for further identification of biomarkers and the development of personalized approaches to maximize its effectiveness. Therefore, pan-cancer studies of target genes are useful for analyzing molecular abnormalities and potential associations in different types of cancer (7). This facilitates advances in combination therapy and individualized therapy.

PSME3, also known as Proteasome Activator Complex Subunit 3, plays a crucial role in regulating essential cellular processes. For instance, PSME3 facilitates the breakdown of the cell cycle inhibitor p21 to stimulate cell proliferation (8). It also serves as a regulator by targeting the mouse double minute 2 homolog/P53 complex (9). PSME3 mediates the secretion of tumor interleukin-1 and necrosis factor-alpha as a transcriptional regulator (10). Furthermore, in pancreatic cancer, PSME3 targets the cellular myelocytomatosis oncogene (c-Myc) to stimulate lactate secretion (11). It also regulates tumor biological functions such as tumor angiogenesis, cell senescence or apoptosis, and lipid and energy metabolism (12–16). For instance, PSME3 participates in angiogenesis by influencing protein kinase (PKA) conversion in the cyclic adenosine monophosphate/PKA signaling pathway (15). Furthermore, it can control the growth of cells with cancer and the onset of BRCA and lung cancer by degrading the steroid receptor co-activators 3, P21, and P53 (8, 9, 17). It regulates energy metabolism in mice by influencing Sirt1-mediated autophagy (12).

The tumor microenvironment (TME) is an active facilitator of cancer progression during tumor growth rather than a silent bystander (18). An in-depth investigation of the dynamic regulatory mechanism of stromal and immune components in the TME and elucidation of the immune phenotype of tumor-immune interactions may provide new cancer treatment targets (19). PSME3 can create a positive feedback cycle with nuclear factor kappa-B (NF-κB), promote the development of colitis and related colon cancer, and mediate the cross-linking between NF-κB and Yes-associated protein pathways (10). Hence,These findings suggest that PSME3 may have a potential role in the immune microenvironment.

However, despite the previous reports on PSME3 in the mentioned cancer types, there hasn’t been a comprehensive pan-cancer study conducted to date. In this study, we conducted a comprehensive investigation aimed at exploring the differential expression of PSME3 in various cancers, its prognostic value, clinical pathological staging, metastasis, and biological functions. We also focused on the role of PSME3 in the tumor immune microenvironment, which was further validated through flow cytometry analysis to gain a deeper understanding of its involvement in immune processes. Additionally, we predicted potential drugs targeting PSME3. Finally, we established the independent prognostic value of PSME3 in LIHC. These research findings provide valuable clues for the development of new targeted therapies.

## 2 Materials and methods

### 2.1 Data preparation and analysis of differential expression

GTEx databases (https://commonfund.nih.gov/GTEx) was used to gather the gene expression information for different tissues. The TCGA transcriptome data were found using UCSC Xena (https://xena.ucsc.edu/) On the expression data and matching tumor types, a log2 transformation and t-test were run. Boxplots were created using the “ggplot” R tool.

### 2.2 PSME3 immunohistochemical staining

The HPA database (https://www.proteinatlas.org/) provides the expression and distribution patterns of approximately 26,000 types of proteins in human tissues and organs. We downloaded immunohistochemistry images of various types of tumor tissues and their corresponding normal tissues from the HPA database.

### 2.3 Survival analysis

Overall Survival (OS) and Recurrence-Free Survival (RFS) were visualized and analyzed using the KMPlot website (https://kmplot.com/analysis/).

### 2.4 Gene set enrichment analysis

The top 100 co-expressed genes in the TCGA dataset were obtained from GEPIA2.0 (http://gepia2.cancer-pku.cn). The PSME3 protein-protein interaction (PPI) network was constructed using the online network tool Search Tool for the Retrieval of Interacting Genes and Proteins (STRING) (https://www.string-db.org/). Enrichment analysis was performed using the R package clusterProfiler (version 3.14.3).

### 2.5 Examination of the immune function of PSME3 in the pan-cancer microenvironment

We calculated immuneScore, stromalScore, and ESTIMATEScore using the Sanegrbox website (http://sangerbox.com/) to evaluate the immune and stromal components. To analyze the association between PSME3 and immune cells, we employed six different immune algorithms, including TIMER, xCell, MCP-counter, CIBERSORT, EPIC, and QuanTIseq. The analysis of immune checkpoints was also conducted using the Sanegrbox website. Additionally, we utilized the SpatialDB online tool to analyze spatial transcriptome data of PSME3 in the mouse brain. We used the TISMO tumor immune network tool (http://tismo.cistrome.org/) to compare gene expression levels before and after cytokine treatment, as well as before and after PDL1 and CTLA-4 treatments in cell lines.

### 2.6 Single-cell sequencing analysis

Using the Tumor Immune Single Cell Center (TISCH) (http://tisch.comp-genomics.org/home/), a single-cell RNA (scRNA)-seq database focused on the TME, we compared the LIHC PSME3 expression in various cell types.

### 2.7 Drug sensitivity of PSME3 in the pan-cancer analysis

We downloaded the NCI-60 compound activity data and RNA-seq from the Genomic of Drug Sensitivity in Cancer (GDSC) (https://www.cancerrxgene.org/) databases. Expression profiling and the examination of PSME3 drug sensitivity in the pan-cancer analysis were performed. The ‘limma’, ‘ggplot2’, and ‘ggpubr’ R packages were used.

### 2.8 Univariate and multivariate cox regression

We conducted univariate Cox regression analysis on liver cancer patients, examining gene expression in relation to overall survival. Multivariate Cox regression was employed to assess independent risk factors within the same cohort. Genes and factors with a false discovery rate (FDR) < 0.05 were deemed significantly associated with patient survival. The outcomes of both univariate and multivariate Cox regression were obtained and visualized using the R package forestplot.

### 2.9 Development of a nomogram

This study employed the Cox regression model and the R package rms to devise an OS prediction nomogram. The nomogram was designed with endpoints set at 1, 2, 3, and 5-year overall survival rates for liver cancer.

### 2.10 Cell culture

The human liver cancer cells (Hut7), human lung cancer cells (A549), human bladder cancer cells (T24), and HEK 293T cells were purchased from the ATCC cell repository. At 37°C and 5% CO2 in a humid incubator, cells were grown in DMEM medium supplemented with 10% heat-inactivated fetal bovine serum, penicillin (100 units/mL) from Gibco, and streptomycin (100 ug/mL) from Gibco.

### 2.11 Plasmid construction and lentivirus transfection

The primer sequences of shRNA were synthesized by Shanghai Biotech. sh1-PSME3 target sequence is 5’-CGTGACAGAGATTGATGAGAA -3’. sh2-PSME3 target sequence is 5’-GCATCTTATCTGGACCAGATT-3’. sh2-PSME3 target sequence is 5’-GCATCTTATCTGGACCAGATT-3’. The PCR instrument was annealed using the combined primers, and the annealing procedures were 37°C (30 min); 95°C (5 min); 95°C (1°C lower per cycle, 1s lower 0.2°C, 20 cycles); 75°C (1°C lower per cycle, 1s lower 0.1°C, 20 cycles); and 12°C (permanent). Finally, the annealed product and vector backbone pLKO.1 were ligated using a high-performance ligase.

Virus packaging was performed in HEK 293T cells according to the ratio pMD2G: psPAX2: plasmid: PEI = 1:2:4:12 (ug:ug:ug:ul). T24 cells were infected at a density of approximately 40%, and virus and medium were added in equal proportions along with 1x of infection reagent PB. Fluid was changed on day 2, and plasmid-resistant drugs were started on day 3 of screening. The pCDNA3.1-Flag-PSME3 overexpression plasmid originates from our laboratory.

### 2.12 Western blotting

Cell lysates were used to obtain total proteins. Proteins were electrotransferred onto polyvinylidene difluoride (PVDF) membranes following SDS-PAGE, and the membranes were sealed with 5% skim milk before being incubated. The membrane was then incubated with Anti-PSME3 antibody (ab180829), Anti-Flag antibody(ab95045), Alpha Tubulin antibody (11224-1-AP), Proteintech, and anti-Actin antibody (ab197345). Incubate for 14–16 hours after dilution at the corresponding ratio. Rabbit secondary antibody (Abcam ab97051) diluted 1:4000 was incubated for 1 h at room temperature, and burst scanning was performed using a high-sensitivity ECL chemiluminescence test kit (Chengdu Gechi Bio 2212ECL013).

### 2.13 Flow cytometry

We prepared cell flow antibodies (dilution ratio 1:100) using Purified anti-human CD276 (B7-H3) antibodies (BioLegend Cat. No. 331602). Resuspended cells were stained on ice for half an hour, washed twice, and fixed using 1% paraformaldehyde. The samples were obtained on the machine using BD Diva software (BD Biosciences). And the data were analyzed using Flowjo 10.

### 2.14 Cell proliferation analysis

To assess the effect of PSME3 silencing on Hut7 cell proliferation, we conducted CCK-8 cell proliferation assays. Cells were seeded in 96-well plates, and CCK-8 reagent was added at different time points (24 hours and 48 hours). Cell proliferation capacity was evaluated by measuring absorbance.

### 2.15 Scratch wound healing assay

Cells were seeded in 6-well culture plates and allowed to grow to a uniform monolayer. Subsequently, a uniform scratch was created in the cell monolayer using a 200μL pipette tip. The ability of cells to heal in the scratched area was photographed, recorded, and compared at different time points.

### 2.16 Data analysis

All experimental data were subjected to statistical analysis using appropriate biostatistical methods, including t-tests and analysis of variance. Experimental results were considered statistically significant when the p-value was less than 0.05.

## 3 Results

### 3.1 Differential expression based on PSME3

**Figure 1:**
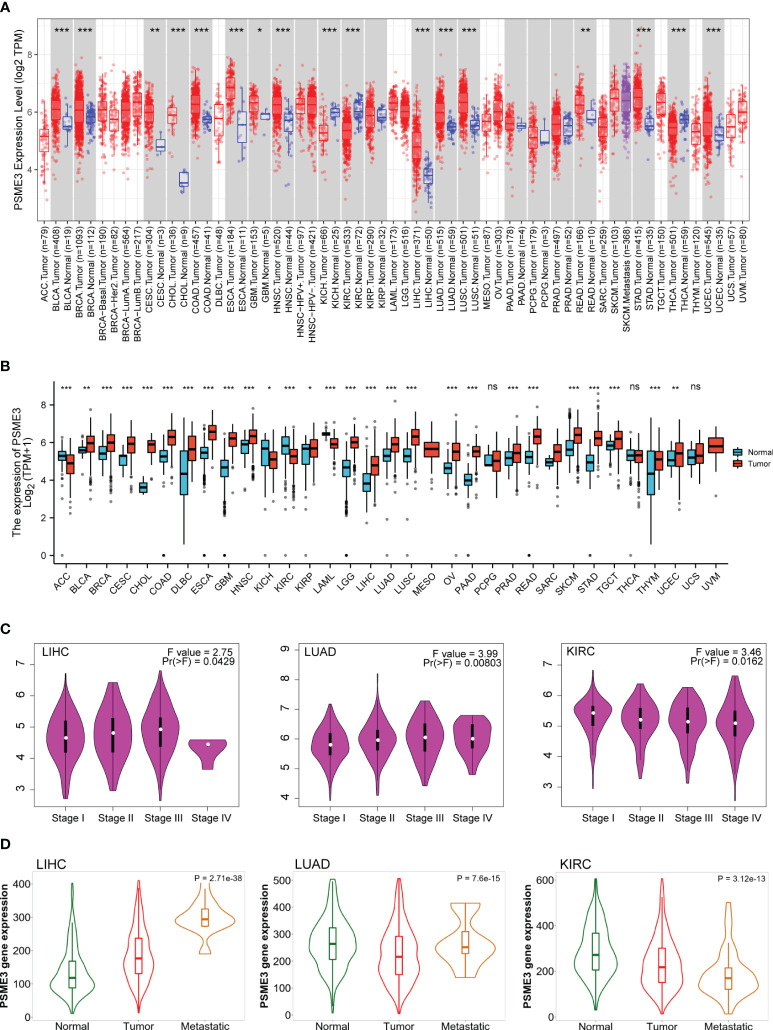
Differential expression of PSME3 **(A)** PSME3 mRNA expression levels in 33 different tumor types derived from TCGA database. Red columns represent cancer samples, and blue columns represent normal samples. *P<0.05, **P<0.01. **(B)** Comparison of PSME3 expression between tumor and normal tissues, combining data from TCGA and GTEx. *P<0.05, **P<0.01, ****P<0.001, NS (no significant differences). **(C)** Violin plots illustrating PSME3 mRNA levels at pathological stages (stages I, II, III, and IV) in various cancers. **(D)** Relationship between PSME3 expression and metastasis in different tumors [log2 transcript per million (TPM) + 1]; only cancers with statistically significant differences between pathological stages are presented.

We conducted an in-depth analysis of RNA sequencing data from the TCGA and GTEx databases. The analysis of the TCGA database reveals a significant upregulation of PSME3 gene mRNA expression in 15 distinct cancer types compared to normal tissues. These cancer types encompass bladder cancer (BLCA), breast cancer (BRCA), cervical squamous cell carcinoma (CESC), cholangiocarcinoma (CHOL), colon adenocarcinoma (COAD), esophageal cancer (ESCA), glioblastoma multiforme (GBM), head and neck squamous cell carcinoma (HNSC), kidney renal clear cell carcinoma (KIRC), LIHC, LUAD, lung squamous cell carcinoma (LUSC), rectum adenocarcinoma (READ), stomach adenocarcinoma (STAD), and uterine corpus endometrial carcinoma (UCEC). Only two cancer types, kidney chromophobe (KICH) and thyroid carcinoma (THCA), exhibit lower PSME3 gene expression when compared to normal tissues (**Figure 1A**).

Considering the limited availability of normal tissue data within the TCGA dataset, we conducted a comprehensive analysis by seamlessly integrating gene expression data sourced from the GTEx database, which offers a larger dataset of paired normal tissues. The combined analysis demonstrates a prominent elevation in PSME3 mRNA expression across several cancer types, including BLCA, BRCA, CESC, CHOL, COAD, Diffuse Large B-Cell Lymphoma (DLBC), ESCA, GBM, HNSC, KICH, Kidney Renal Papillary Cell Carcinoma (KIRP), Acute Myeloid Leukemia (LAML), Low-Grade Glioma (LGG), LIHC, LUAD, LUSC, Ovarian Cancer (OV), Pancreatic Ductal Adenocarcinoma (PAAD), PRAD, READ, Skin Cutaneous Melanoma (SKCM), STAD, Testicular Germ Cell Tumor (TGCT), Thymoma (THYM), and UCEC. Only in adrenocortical carcinoma (ACC), kidney renal clear cell carcinoma (KIRC), and kidney chromophobe (KICH) do we observe a contrasting trend (**Figure 1B**).

We conducted a further analysis of the expression patterns of PSME3 in different cancers concerning pathological staging and metastasis. The data indicates a correlation between the expression of PSME3 and the pathological staging as well as tumor metastasis in cases of LUAD, LIHC, and KIRC (**Figures 1C, D**).

**Figure 2:**
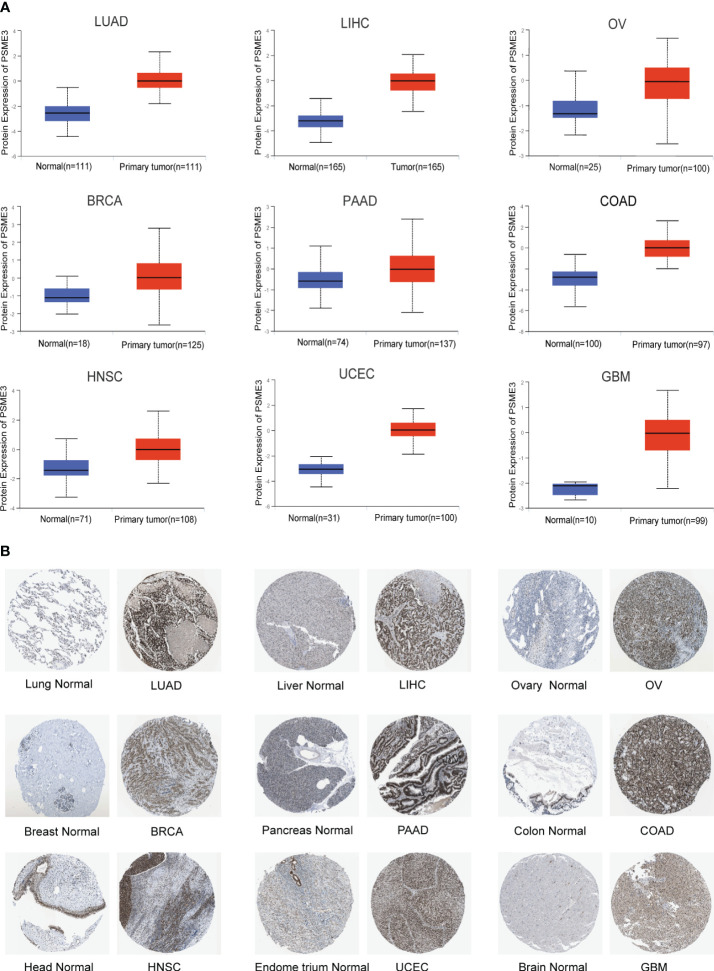
**(A)** Differential expression of PSME3 protein in various tumor and normal tissues from the UALCAN database. **(B)** Immunohistochemical images comparing PSME3 protein expression in normal (left) and tumor (right) tissues.

In the previously mentioned tumors exhibiting mRNA level differences, we proceeded with an analysis of PSME3 protein expression using the UALCAN database. The results revealed a significant increase in PSME3 protein expression in LIHC, UCEC, BRCA, OV, HNSC, PAAD, GBM, COAD, and LUAD. These findings are consistent with our previous research outcomes (**Figure 2A**). Furthermore, immunohistochemical data from the HPA database provided additional support for our discoveries (**Figure 2B**). These findings may contribute to a deeper understanding of the biological functions of PSME3 in various cancers and shed light on its potential clinical diagnostic value.

### 3.2 The prognostic significance of PSME3 expression in pan-cancer

**Figure 3:**
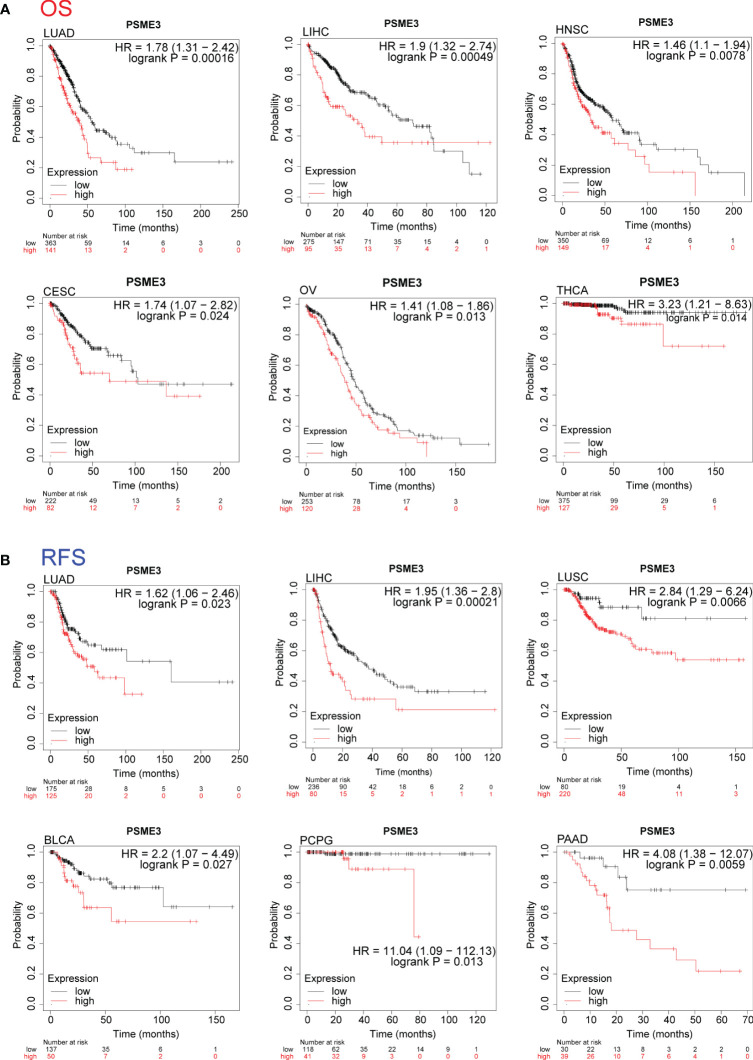
**(A)** Kaplan-Meier analysis of the relationship between high and low expression of PSME3 and OS(HR>1, P<0.05). **(B)** Kaplan-Meier analysis of the relationship between high and low expression of PSME3 and RFS (HR>1, P<0.05).

We conducted a pan-cancer analysis using the Kaplan-plot tool to assess the prognostic significance of PSME3. The results revealed that in LIHC, HNSC, LUAD, OV, THCA, and CESC, high PSME3 expression was associated with poorer OS, with a hazard ratio (HR) greater than 1 (**Figure 3A**; P < 0.05). However, in gastric cancer STAD (**Supplementary Figure 1A**), high PSME3 expression was correlated with better OS. Furthermore, in HNSC, LIHC, BLCA, LUAD, LUSC, and PCPG, high PSME3 expression was linked to worse RFS (**Figure 3B**; HR > 1, P < 0.05), while in STAD, ESCA, KIRC, and OV, high PSME3 expression was associated with better RFS (**Supplementary Figure 1B**).

In summary, the PSME3 particularly in LIHC and LUAD, exhibits significantly elevated expression levels. This elevated expression is associated with poor clinical prognosis and cancer progression, indicating its potential clinical utility. Further research into the biological functions of PSME3 will contribute to the development of more effective cancer treatment strategies.

### 3.3 The biological functions of PSME3

**Figure 4:**
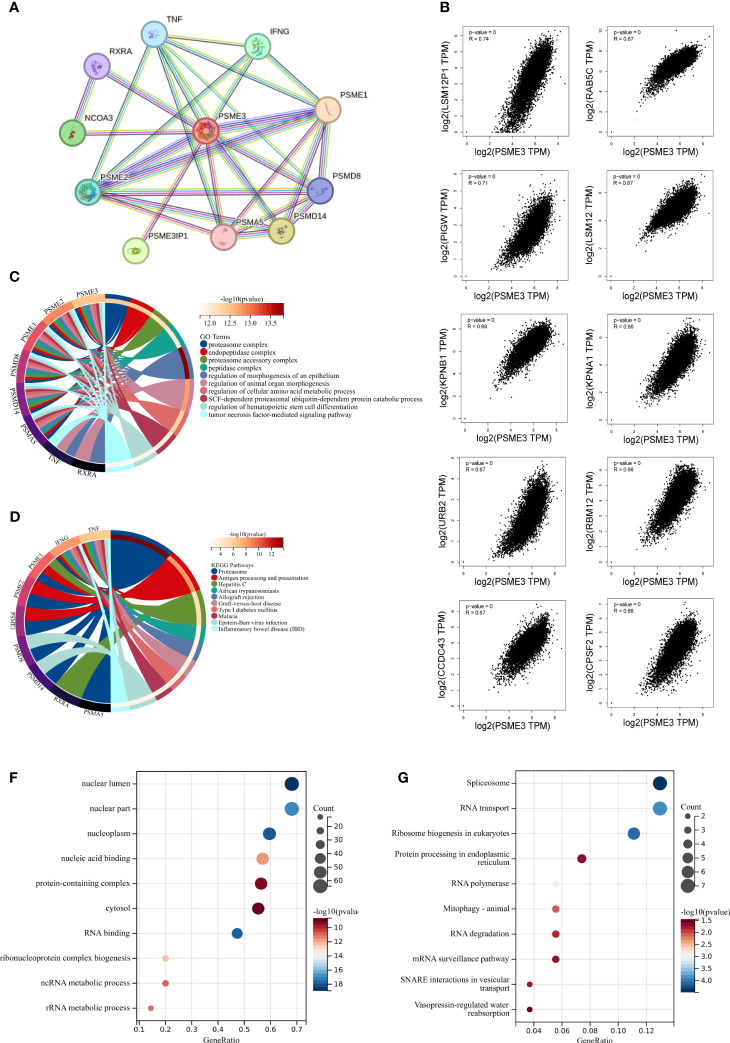
The enrichment analysis of PSME3 in pan-cancer: **(A)** Interaction network of PSME3’s interacting proteins as retrieved using the protein-protein interaction search tool (STRING). **(B)** Correlation between PSME3 and the top 10 co-expressed genes. **(C)** Functional enrichment analysis of GO pathways associated with PSME3 and its interacting proteins. **(D)** Functional enrichment analysis of KEGG pathways associated with PSME3 and its interacting proteins. **(E)** Functional enrichment analysis of GO pathways associated with PSME3 and its co-expressed genes. **(F)** Functional enrichment analysis of KEGG pathways associated with PSME3 and its co-expressed genes.

Our study commenced by analyzing proteins that interact with PSME3 and exploring co-expressed genes. Subsequently, we conducted functional enrichment analysis. In the STRING database, we identified ten proteins known to interact with PSME3: PSME1, PSME2, PSMA5, PSMD8, PSMD14, PSMEIP1, NCOA3, RXRA, TNF, and IFNG. These proteins collectively constitute a PPI network (**Figure 4A**). Furthermore, we validated the co-expression patterns of PSME3 using GEPIA 2. This analysis unveiled the top 100 genes closely associated with PSME3 (**Supplementary Table 1**), with the top 10 genes listed as follows: LSM12P1 (R = 0.74), PIGW (R = 0.71), LSM12 (R = 0.67), CCDC43 (R = 0.67), RAB5C (R = 0.67), KPNA1 (R = 0.66), RABM12 (R = 0.66), CPSF2 (R = 0.66), URB2 (R = 0.67), and KPNB1 (R = 0.69) (**Figure 4B**).

Through Go and KEGG functional enrichment analysis, our research has unveiled the pivotal roles played by PSME3 and its interacting proteins in a multitude of biological processes. These processes encompass protein degradation, antigen processing and presentation, immune rejection reactions, autoimmune diseases and inflammation, regulation of hematopoietic stem cell differentiation, and amino acid metabolism (**Figures 4C, D**). Additionally, the functional enrichment analysis of the top 100 genes co-expressed with PSME3 has revealed a wide spectrum of cellular processes, including RNA processing, protein synthesis, organelle functions, and signal transduction pathways (**Figures 4E, F**). These findings underscore the multifunctionality of PSME3 in cell biology, immunology.

### 3.4 Immune infiltration and immune cell analysis based on PSME3

**Figure 5:**
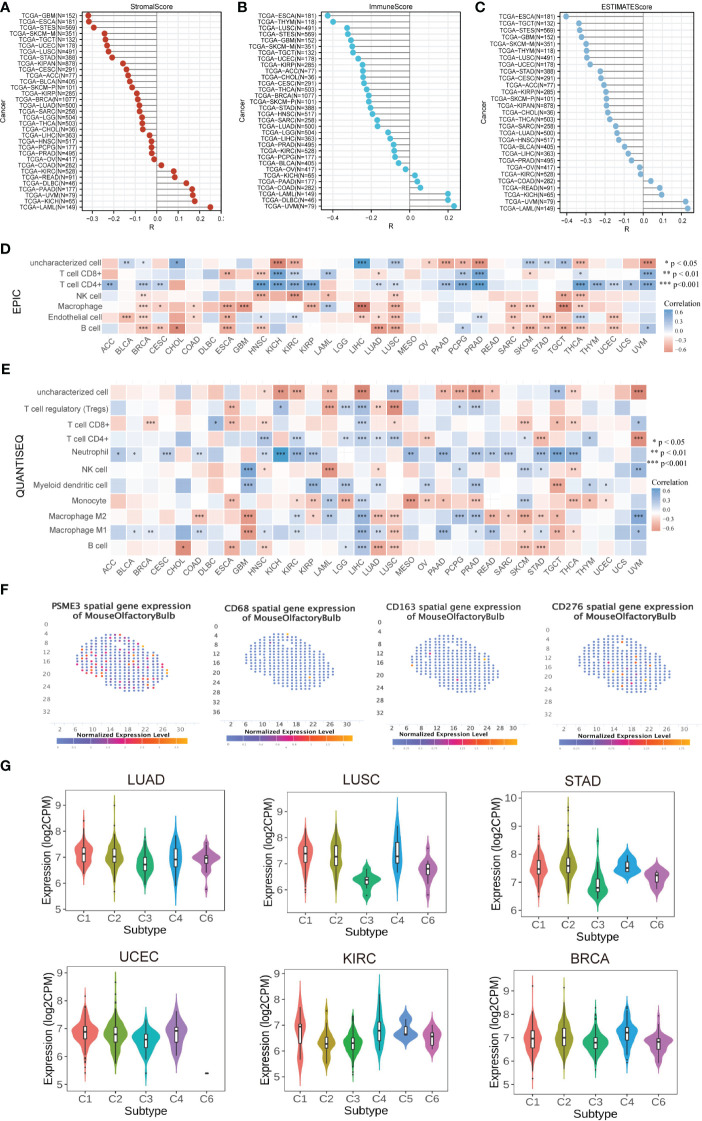
Correlation and Immune Cell Infiltration Analysis **(A)** Stick map illustrating the correlation between PSME3 and StromalScore. **(B)** Stick map illustrating the correlation between PSME3 and ImmuneScore. **(C)** Stick map illustrating the correlation between PSME3 and ESTIMATEScore. **(D)** Immune cell infiltration analysis conducted using EPIC. **(E)** Immune cell infiltration analysis conducted using QuanTiseq. *P<0.05, **P<0.01, NS (no significant differences). **(F)** Spatial transcription sections displaying the spatial expression patterns of PSME3, CD68, CD276, and CD163 markers. Dot colors represent the expression levels of the markers. **(G)** Correlations between PSME3 and immune subtypes were analyzed with the TSIDB online tool.

Research on PSME3 within the domain of tumor immunity has been relatively limited in scope. However, given its potential significance in this field, there is a critical need for further exploration and expansion of PSME3 research. As immune infiltrating cells play a pivotal role in cancer development, we conducted an analysis to determine the estimated score, immune score, and stromal score of PSME3 in various cancers (**Figures 4E, F**). It’s worth noting that in specific cancer types such as COAD, KIRC, READ, DLBC, PAAD, UVM, KICH, and LAML, PSME3 exhibited a positive correlation with the Stromal Score. Additionally, a positive correlation was observed between PSME3 and Immune Score in COAD, DLBC, PAAD, UVM, KICH, and LAML. Furthermore, the Immune Scores in COAD, READ, PAAD, UVM, KICH, and LAML displayed a positive correlation with the estimates (**Figures 5A–C**).

Furthermore, we employed six different techniques to assess immune cell involvement, aiming to elucidate the relationship between PSME3 and immune cells. These methods included EPIC (**Figure 5D**), QuanTIseq (**Figure 5E**), CIBERSORT (**Supplementary Figure 2A**), TIMER (**Supplementary Figure 2B**), XCell (**Supplementary Figure 2C**), and MCP-counter (**Supplementary Figure 2D**). Notably, higher levels of PSME3 exhibited a robust positive correlation with neutrophils, B cells, CD4 T cells, CD8 T cells, and M2 macrophages in cancers such as HNSC, KIRC, KICH, PRAD, and UVM. To further validate these findings, we conducted a spatial transcriptional analysis in the mouse brain, confirming the spatial co-expression of PSME3 with the M2 macrophage biomarkers CD68 and CD163. The geographical distribution patterns of PSME3, CD68, and CD163 markers showed significant overlap (**Figure 5F**).

Furthermore, results from immune subtype analysis indicated that elevated expression of PSME3 was associated predominantly with the C2 subtype in BLCA, UCEC, STAD, and LUAD, suggesting a primary association with IFN-gamma. Meanwhile, the increased presence of PSME3 in the C4 subtype of LUSC and KIRC implied an association with lymphocyte exhaustion (**Figure 5G**).

### 3.5 Association between PSME3 and immune checkpoint

**Figure 6:**
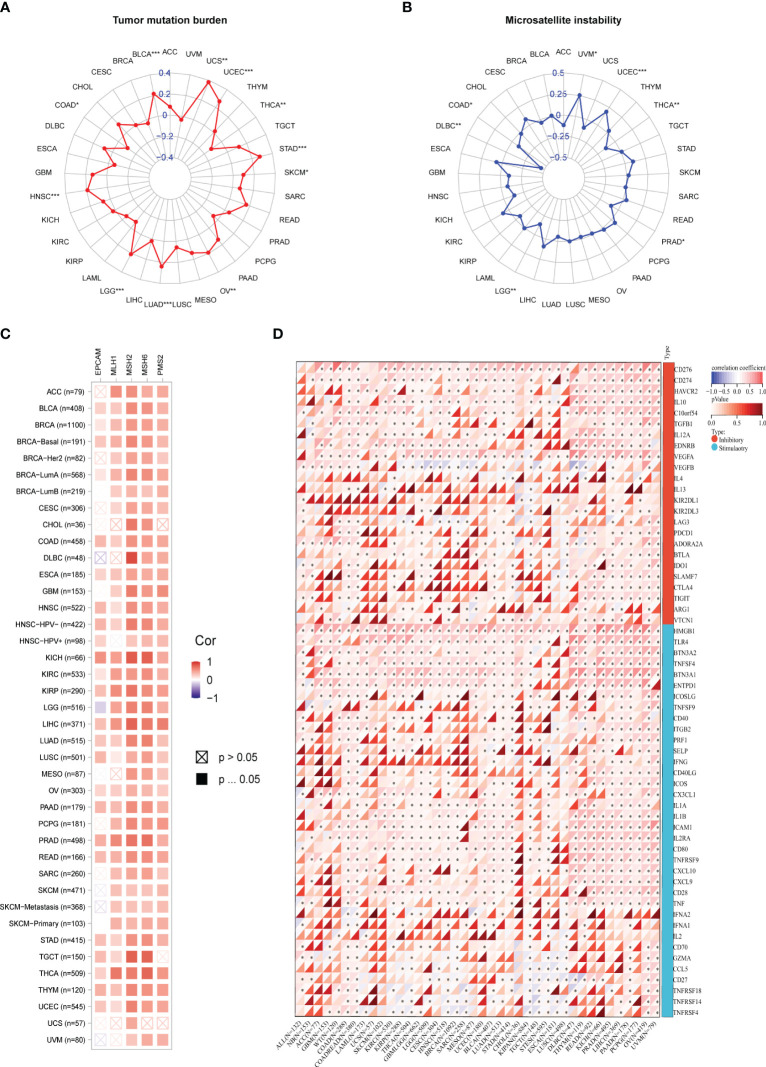
**(A)** Correlation Between PSME3 mRNA Expression and TMB. **(B)** Correlation Between PSME3 mRNA Expression and MSI. **(C)** Heatmap illustrating the expression of PSME3 and MMR pathway genes. **(D)** Heatmap depicting the relationship between immune checkpoint genes and PSME3 expression in pan-cancer analysis. *P < 0.05, **P < 0.01, ***P < 0.001.

Antitumor immunity is a powerful predictor of immunotherapy response associated with MSI (Microsatellite Instability), TMB (Tumor Mutational Burden) (20). Immune checkpoint inhibitor therapy’s effects can be detected by MSI-H/dMMR and a high TMB (TMB-H) (21). In our study, we utilized the Pearson correlation coefficient to assess the association between the PSME3 gene expression levels and TMB as well as MSI. We looked into the correlation between PSME3 expression levels and those of MSI, TMB. The expression levels of PSME3 were positively correlated with TMB in LUAD, UCEC, BLCA, UCEC, STAD, SKCM, OV, LGG, and HNSC; this was negatively correlated with TMB in THCA (**Figure 6A**). Furthermore, the expression of PSME3 was negatively correlated with MSI in UCEC and UVM while being positively correlated with MSI in COAD, DLBC, LGG, PRAD, and THCA (**Figure 6B**). Based on the association between PSME3 expression and the mutation markers TMB and MSI, we further investigated the link between PSME3 expression and mature MMR genes. From the results, in 33 cancer types (excluding LGG), PSME3 was positively correlated with MMR gene expression (**Figure 6C**).

Immunological checkpoints (ICPs) represent the most promising targets for tumor immunotherapy since they regulate the entry of immune cells into TME (21). When investigating the relationship between immune checkpoint expression and PSME3, we observed that among the 33 cancers, CD276 and CD274 exhibited the highest positive correlation with PSME3, followed by VEGFA, HMGB1, THR4, BTNA2, and ENTDD1 (**Figure 6D**). Spatial transcriptome results also indicated an overlap between CD276 and PSME3 (**Figure 5F**). In summary, the correlation between PSME3 and immune checkpoint expression offers crucial insights into the intricate dynamics within the tumor microenvironment. Continued research in this field could lead to significant progress in personalized cancer treatment strategies.

### 3.6 To validate the involvement of PSME3 in the regulation of CD276

**Figure 7:**
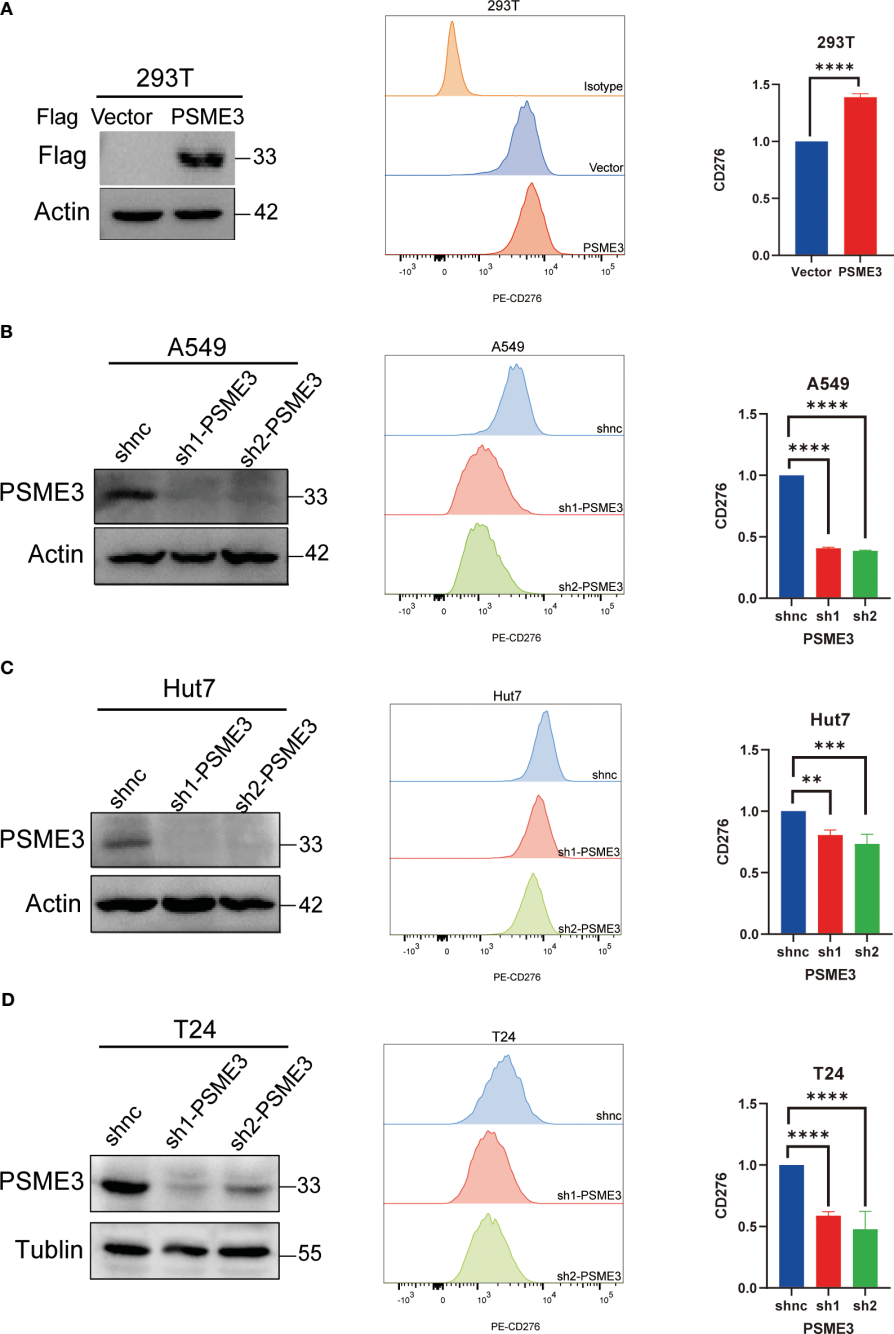
**(A)** CD276 Expression with PSME3 Overexpression in 293T Cells. **(B)** CD276 Expression with PSME3 Knockdown in A549 Cells. **(C)** CD276 Expression with PSME3 Knockdown in Hut7 Cells. **(D)** CD276 Expression with PSME3 Knockdown in T24 Cells. Left: Western blot results; Center: Flow cytometry histograms; Right: Quantified data in a bar graph. *P<0.05, **P<0.01, ****P<0.001.

To further confirm the association between PSME3 and immune checkpoints, we conducted flow cytometry analysis after modulating PSME3 expression in 293T cells (**Figure 7A**) and silencing PSME3 in liver cancer Hut7, lung adenocarcinoma A549, and bladder cancer T24 cells (**Figures 7B-D**). The experimental findings unequivocally demonstrated that PSME3 plays a positive regulatory role in CD276 expression. Intervening to reduce the expression of CD276 by targeting PSME3 is a promising treatment strategy that may contribute to enhancing the efficacy of immunotherapy.

### 3.7 Immunotherapy response and sensitive medication prediction

**Figure 8:**
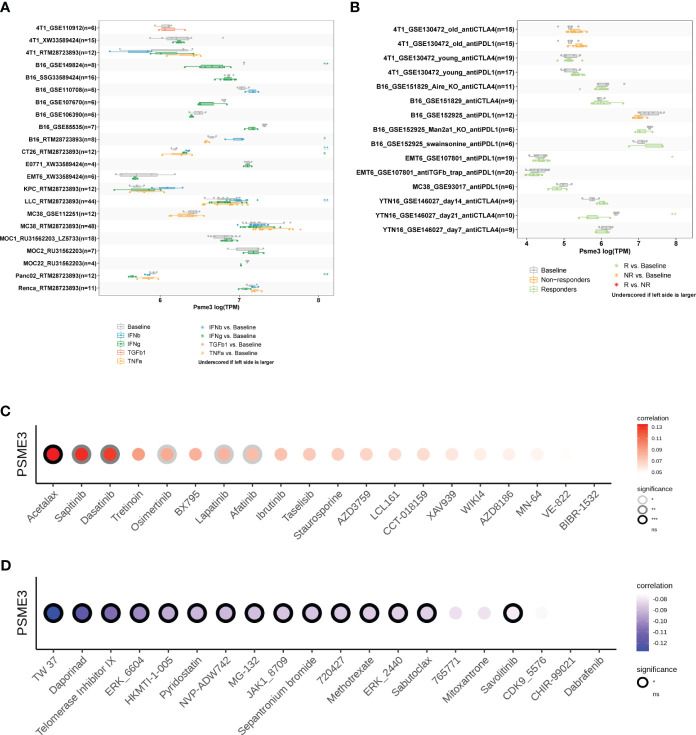
**(A)** Multiple boxplots illustrate the expression of PSME3 in cancer cell lines before and after cytokine treatment, as obtained from the TISMO web tool. Significance levels are indicated as follows: *, **, and *** represent P values of < 0.05, < 0.01, and < 0.001, respectively. **(B)** Multiple boxplots depict the expression of PSME3 in cancer cell lines before and after PDL1 or CTLA4 treatment, as obtained from the TISMO web tool. Significance levels are denoted as follows: *, **, and *** denote P values of < 0.05, < 0.01, and < 0.001, respectively. Based on drug sensitivity analysis in GDSC (Genomics of Drug Sensitivity in Cancer), the following are the top 15 drugs that are positively correlated **(C)** and negatively correlated **(D)** with PSME3 expression, as shown in the figure. *P<0.05, **P<0.01, NS (no significant differences).

We initiated our study by employing the TISMO network tool, which revealed that cytokine therapy, particularly interferons (IFNβ and IFNγ), exhibited promising therapeutic effects in mouse models with high PSME3 expression, especially in various cancer types such as lung cancer, breast cancer, melanoma, and colorectal cancer (**Figure 8A**). Additionally, our observations indicated that the expression levels of PSME3 can predict the response to immunotherapy with PDL1 and CTLA4 in gastric adenocarcinoma and breast cancer in mouse models (**Figure 8B**). These findings provide crucial insights into the potential role of PSME3 in immunotherapy and its association with treatment responses, offering valuable information for future research and the development of immunotherapeutic strategies.

Acetalax, sapitinib, and dasatinib were the top three medications that were positively linked with PSME3 expression, according to analyses of correlations between PSME3 expression and drug sensitivity based on the GDSC dataset (**Figure 8C**). Contrarily, the top three medications that were adversely linked with PSME3 expression were TW 37, daporinad, and telomerase inhibitor IX (**Figure 8D**).

### 3.8 PSME3 as an independent predictor in LIHC

**Figure 9:**
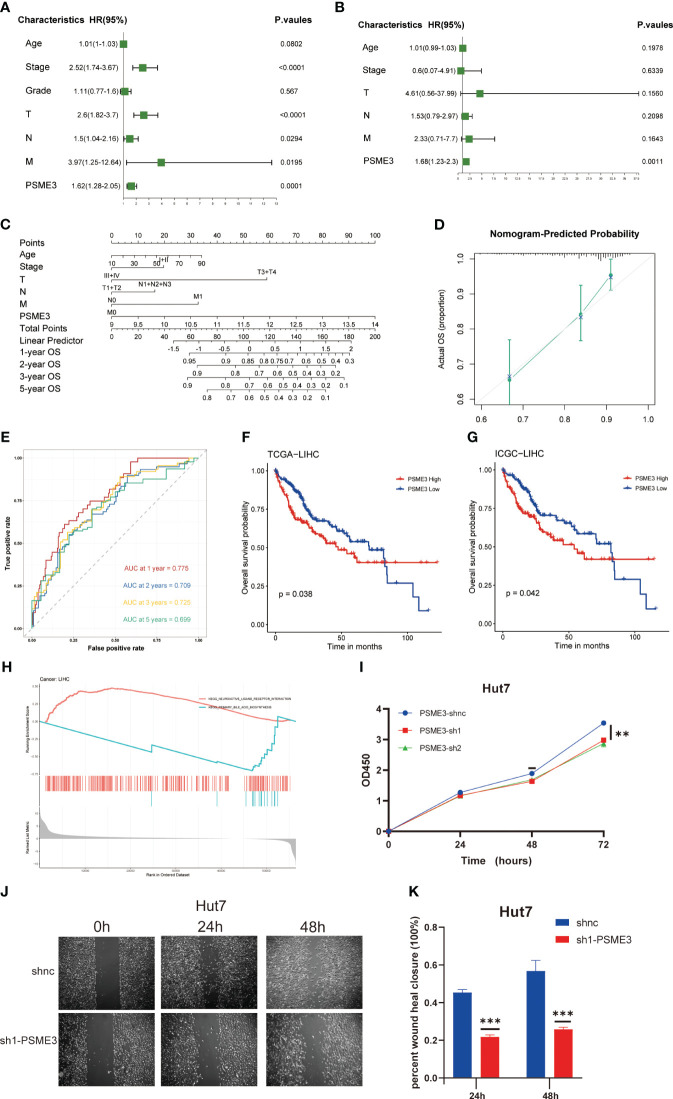
PSME3 as an independent predictor in LIHC. **(A, B)** Prognostic implications of PSME3 in liver carcinoma through univariate and multifactorial COX analysis. **(C)** Construction of a nomogram utilizing PSME3 expression. **(D)** Nomogram correction analysis diagram. **(E)** In the TCGA liver cancer cohort, calibration plots illustrating the 1-year, 2-year, 3-year, and 5-year OS probabilities were displayed (*p<0.05; **p<0.01; ***p<0.001; ****p<0.0001; ns p>0.05). Independent survival prognosis analysis of PSME3 in TCGA **(F)** and ICGC **(G)** datasets. **(H)** GSEA was performed with the KEGG signature of PSME3 in LIHC. Different color curves represent different functions or pathways. The peaks of the rising and falling curves indicate positive and negative regulation of PSME3, respectively. **(I)** Evaluation of PSME3 expression in the Hut7 liver cancer cell line using the CCK-8 assay. **(J, K)** Bar chart representing the quantified data of PSME3 expression in the Hut7 liver cancer cell line following scratch assay. *P < 0.05, **P < 0.01, ***P < 0.001 compared to the control group.

Our previous findings indicate a close correlation between high PSME3 expression and the progression and prognosis of LIHC, with a significant role in liver cancer immunity. For this reason, we proceeded to explore the biological functions and independent prognostic value of of PSME3 in LIHC. We identified PSME3 as an independent prognostic indicator in LIHC through both univariate and multivariate COX regression analyses. (**Figures 9A, B**). Additionally, we constructed a PSME3-based nomogram to better assist in evaluating patient prognosis in clinical practice (**Figure 9C**). The area under the curve (AUC) for the 1-year, 2-year, 3-year, and 5-year OS in the line chart were 0.775, 0.709, 0.725, and 0.699, respectively (**Figure 9D**). Moreover, calibration curves were generated to evaluate the performance of the line chart (**Figure 9E**). These curves demonstrated a close proximity between the predictive curve of the model and the ideal curve.These results indicate a robust predictive performance.

Meanwhile, we conducted independent survival prognosis analyses in TCGA and ICGC databases to further confirm the predictive capacity of high PSME3 expression for a poorer prognosis in liver cancer patients (**Figures 9F, G**). Furthermore, clinical relevance indicated an association between high PSME3 expression and specific tumor staging (T1-T4/I/II/III/IV stages), irrespective of age (**Supplementary Figure 3A**).

To better elucidate the potential mechanisms through which PSME3 influences patient prognosis, we analyzed single-cell sequencing data and observed a noteworthy correlation between PSME3 and CD4Tconv, CD8T, CD8Tex and Tprolif (**Supplementary Figure 3B**). Additionally, KEGG pathway enrichment analysis revealed significant associations between PSME3 expression and pathways related to neuroactive ligand receptor interactions and primary bile acid biosynthesis (**Figure 9H**).

Finally, we conducted initial validation of the general functions of PSME3 in liver cancer. Both CCK-8 assays and wound healing assays provided confirmation of PSME3’s impact on Hut7 cell proliferation. The results were unequivocal, demonstrating that the knockdown of PSME3 significantly decreased the proliferation capacity of Hut7 cells compared to the control group. Additionally, the healing capacity of the PSME3 knockdown group remained notably inferior to that of the control group (**Figures 9I-K**).

## 4 Discussion

Immune checkpoint inhibitors (ICIs) play a pivotal role in cancer immunotherapy by modulating immune responses to help restore the body’s immune response against tumors. Despite the significant achievements of immune checkpoint inhibitors like PD-1/PD-L1 inhibitors in some cancer treatments, resistance to therapy in some patients and inconsistent efficacy remain challenges. Therefore, the search for new immune checkpoints or biomarkers to predict the effectiveness of immunotherapy becomes particularly important. In our research, we have demonstrated that PSME3 could serve as a promising prognostic biomarker, especially in the context of future cancer immunotherapy, holding potential significance.

Finding biomarkers useful for broad-spectrum cancer diagnosis is aided by the analysis of differential gene expression. Protein analysis offers compelling evidence for the identification of prognostic biomarkers for early diagnosis (22, 23). Our research results indicate that abnormal PSME3 mRNA expression is observed in nearly all types of cancer within the TCGA dataset. Importantly, our immunohistochemical analysis provides further confirmation, strongly supporting the high consistency between PSME3 protein levels and mRNA expression across various cancer types. Furthermore, we find associations between PSME3 expression and tumor staging as well as metastasis in kidney, liver, and lung cancers. Most significantly, high PSME3 expression is significantly correlated with OS and RFS.

Previous reports have also mentioned the abnormal expression of PSME3 in various types of cancer, including thyroid cancer, head and neck cancer, and osteosarcoma, which is consistent with our research findings (24–29). The investigators found that the prognosis of patients with PSME3-negative tumor tissue was significantly better than that of the PSME3-positive group (30); Roessler M. et al. identified PSME3 as a new serum tumor marker for colorectal cancer based on mass spectrometry analysis as early as 2006 (31). It further indicated that PSME3 is a potential prognostic biomarker for various cancers.

PSME3 is a proteasomal activator that functions by activating the 20S proteasome to degrade proteins (32–34). Our functional enrichment analysis has revealed that PSME3 is involved in multiple biological functions, such as protein degradation, lipid metabolism, immune responses, and stem cell regulation, among others. These findings align with previous research, for example, PSME3 targets several key proteins involved in cell cycle regulation, including the cell cycle arrest protein P21, and two cell cycle-dependent kinase inhibitors, P16 and P14 (8, 35). Additionally, the deficiency of PSME3 disrupts the function of MDM2, leading to the stabilization of p53 and the upregulation of p21, consequently resulting in cell cycle arrest (9). PSME3 regulates autophagy and hepatocyte lipid metabolism by affecting Sirt1 (12).

The immune microenvironment plays a significant role in the TME (36). Immune cell invasions can either inhibit or promote the development and progression of tumors, including tumor escape, invasion, metastasis, and treatment (37, 38). Our results indicated an association between elevated PSME3 expression and the presence of CD8T, and M2 macrophages. Our research also unveiled the association of PSME3 with immune checkpoints, with experimental validation demonstrating that PSME3 positively regulates CD276 expression. This suggests that PSME3 might be a potential therapeutic target in immunotherapy.

Additionally, we predicted sensitive drugs targeting PSME3, providing crucial clues for future treatment strategies. In further analyses, we discovered a close correlation between high PSME3 expression and adverse clinical outcomes and cancer progression in LIHC. The independent prognostic value of PSME3 in LIHC was validated, underscoring its significance in assessing the prognosis of liver cancer patients. We also constructed a prognostic model based on PSME3, which exhibited high predictive performance. Finally, through experimental validation, we confirmed the impact of PSME3 on liver cancer cell proliferation and wound healing, further supporting its biological function in liver cancer.

In summary, our research revealed the multifaceted role of PSME3 in cancer biology, immune regulation, and clinical prognosis, providing crucial insights for the development of personalized cancer treatment strategies and immunotherapy research. PSME3 holds the potential to become a significant target in future cancer therapies, but further research is needed to elucidate its detailed mechanisms and application prospects.

## 5 Conclusion

PSME3 emerges as a key factor positively regulating the immune checkpoint CD276 in various cancers, including LIHC, LUAD, and BLCA. Therefore, it holds the potential to become a promising target for immunotherapy. Additionally, PSME3 has been confirmed as an independent prognostic factor in LIHC, impacting not only immune regulation but also aspects like liver cancer cell proliferation and wound healing. These discoveries provide crucial insights for the development of future cancer treatment strategies, with the potential to enhance the survival rates and overall quality of life for cancer patients.

## Data availability statement

The original contributions presented in the study are included in the article/**Supplementary Material**. Further inquiries can be directed to the corresponding author.

## Author contributions

CD: Conceptualization, Data curation, Methodology, Validation, Visualization, Writing – original draft, Writing – review & editing. YG: Supervision, Validation, Writing – review & editing. YY: Formal Analysis, Methodology, Writing – review & editing. XG: Conceptualization, Funding acquisition, Resources, Supervision, Writing – review & editing.

## Supplementary material

Supplementary Figure 1
**(A)** Kaplan-Meier analysis of the relationship between high and low expression of PSME3 and OS( HR<1, P<0.05 ). **(B)** Kaplan-Meier analysis of the relationship between high and low expression of PSME3 and RFS ( HR<1, P<0.05 ).

Supplementary Figure 2 Immune cell infiltration was assessed using various methods, including cell-type identification by estimating the relative subset of RNA transcripts (CIBERSORT) **(A)**, TIMER **(B)**, xCell **(C)**, and MCP-counter **(D)**. The results are presented in the form of a heatmap.

Supplementary Figure 3 The clinical correlation analysis of PSME3 with different ages and pathological stages. Single-cell analysis based on the LIHC-GSE98638 dataset: (left:) Provides an overview of the distribution of CD4Tconv, CD8T, CD8Tex, Tprolif, and Treg cells at the single-cell level. (center) Illustrates the expression distribution of PSME3 in LIHC. (right) Presents a violin plot depicting the single-cell expression profile based on PSME3.
