# Supplementary figures and images for: Comprehensive analysis of PSME3: from pan-cancer analysis to experimental validation
Source: Front Immunol. 2024 Jan 19;15:1295693. doi: 10.3389/fimmu.2024.1295693 (PMC10834762; doi:10.3389/fimmu.2024.1295693)

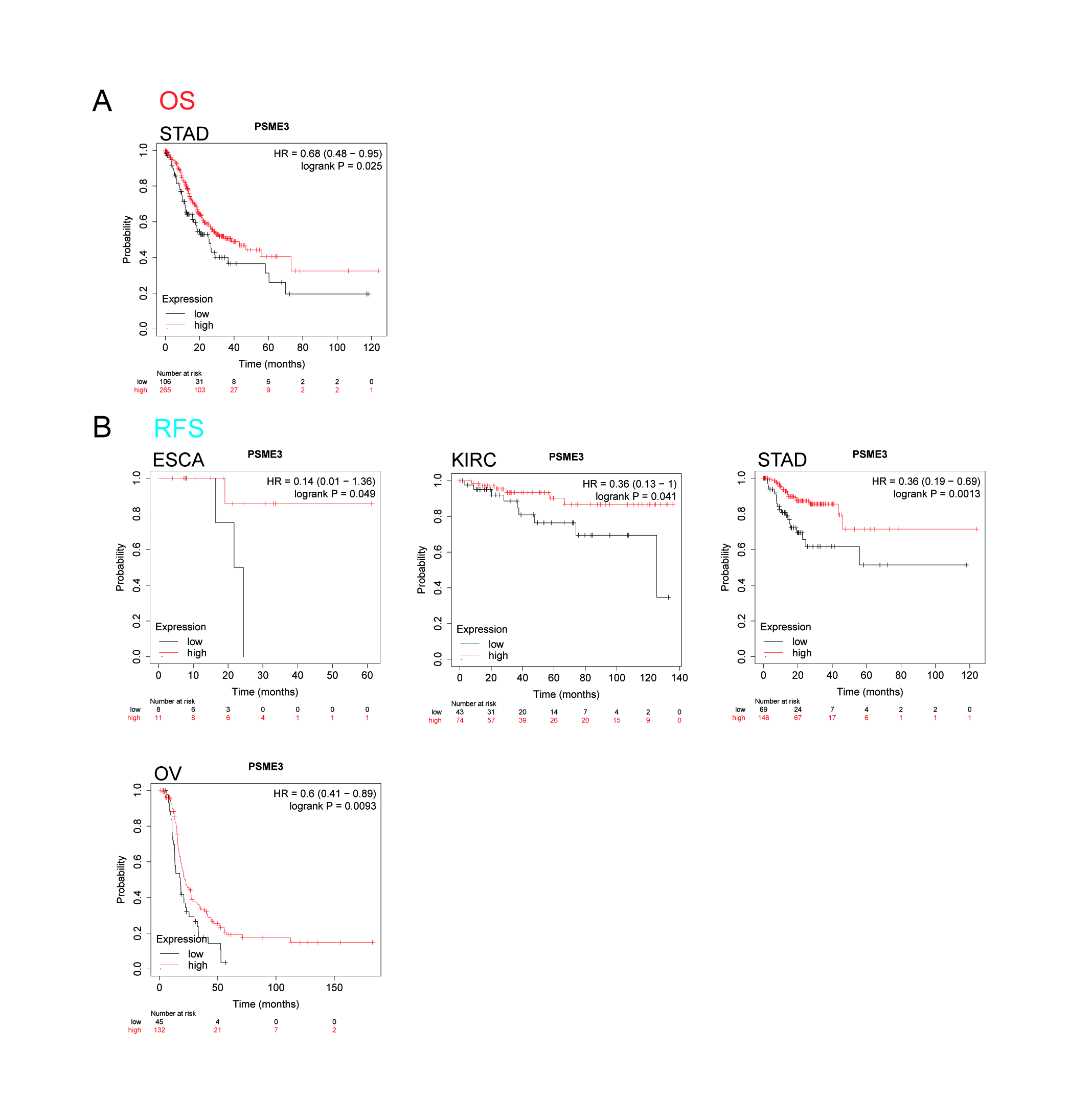

Supplement: Supplementary Figure 1 — (A) Kaplan-Meier analysis of the relationship between high and low expression of PSME3 and OS( HR<1, P<0.05 ). (B) Kaplan-Meier analysis of the relationship between high and low expression of PSME3 and RFS ( HR<1, P<0.05 ). [file Image_1.tif]

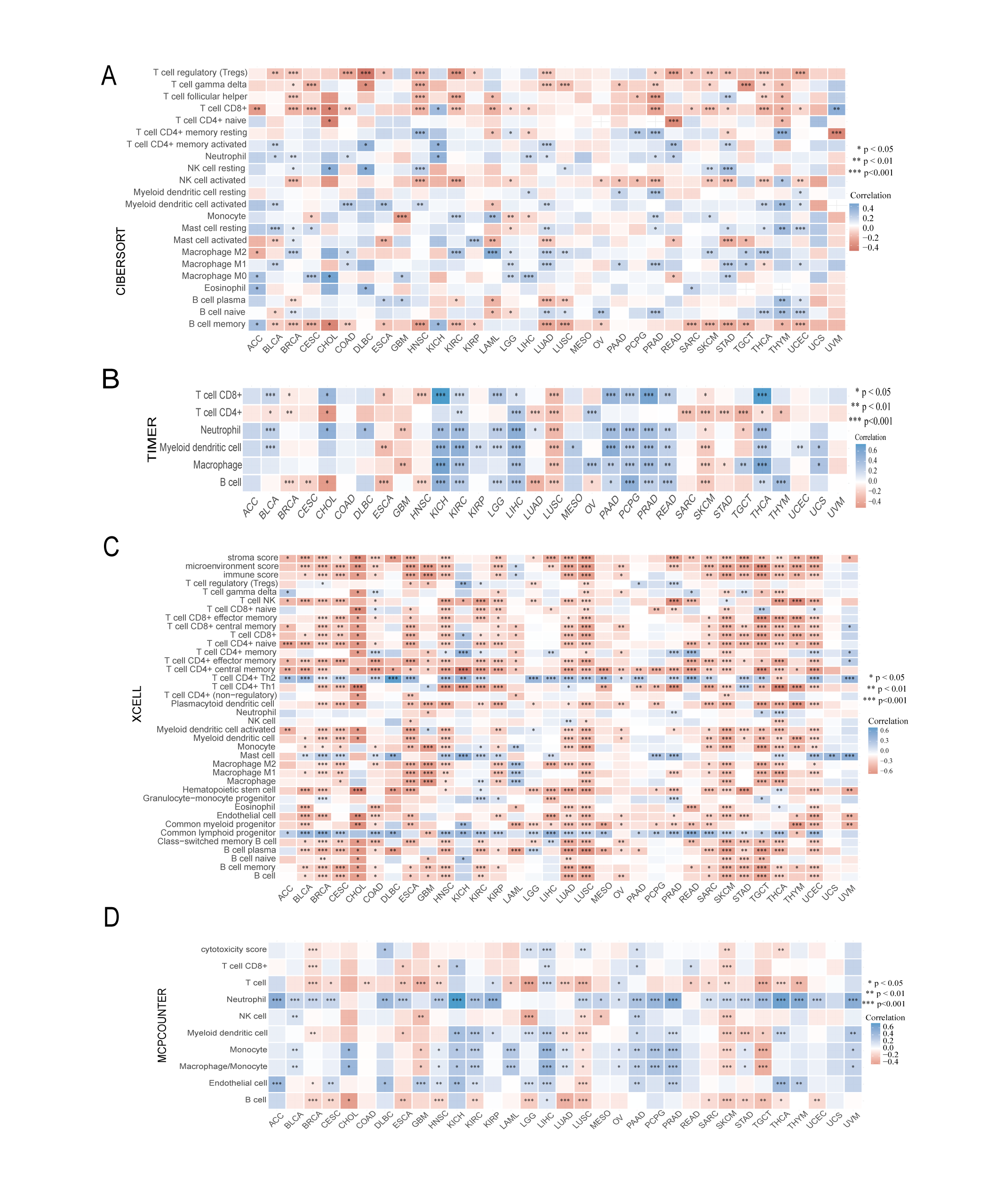

Supplement: Supplementary Figure 2 — Immune cell infiltration was assessed using various methods, including cell-type identification by estimating the relative subset of RNA transcripts (CIBERSORT) (A), TIMER (B), xCell (C), and MCP-counter (D). The results are presented in the form of a heatmap. [file Image_2.tif]

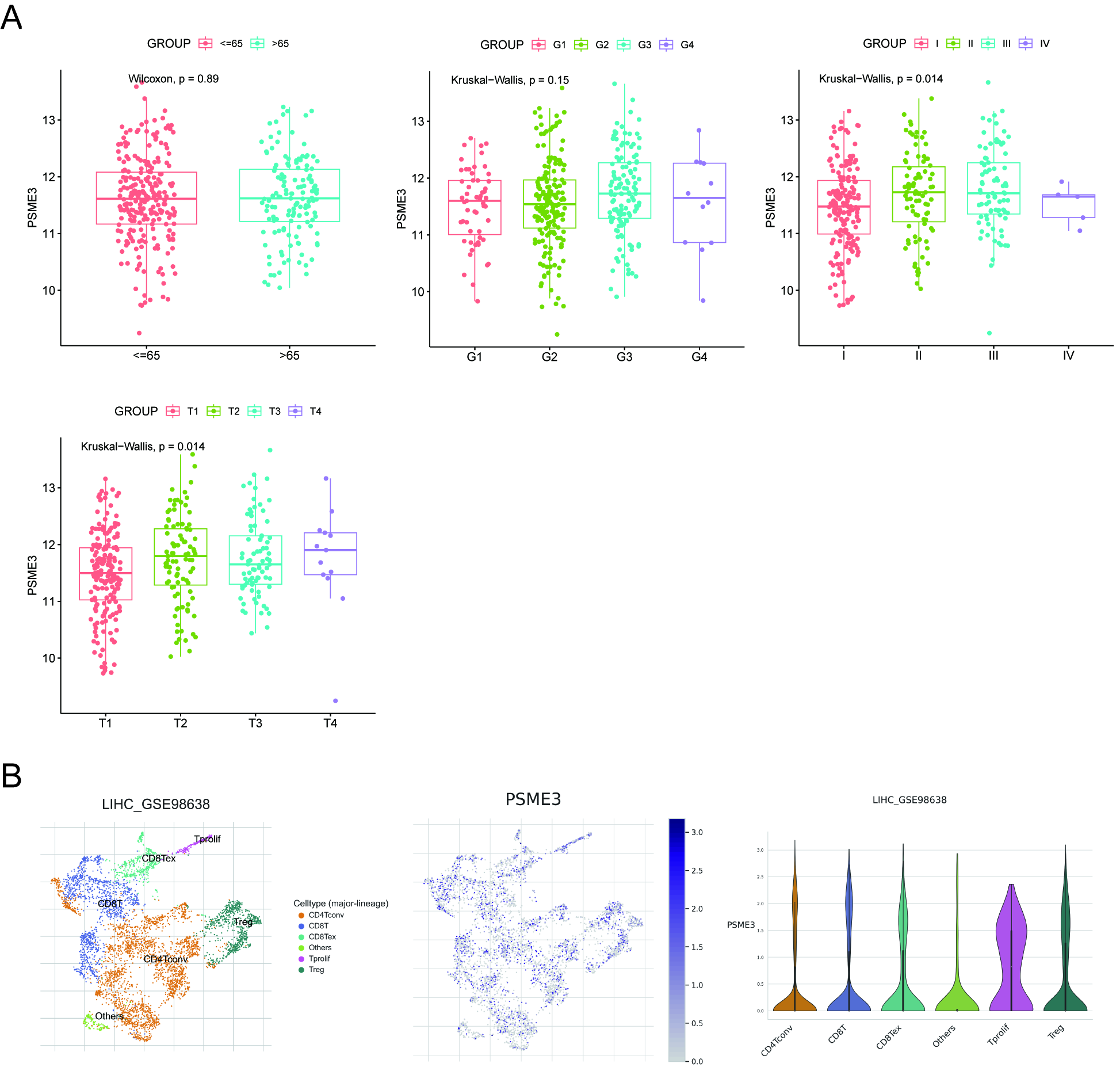

Supplement: Supplementary Figure 3 — The clinical correlation analysis of PSME3 with different ages and pathological stages. Single-cell analysis based on the LIHC-GSE98638 dataset: (left:) Provides an overview of the distribution of CD4Tconv, CD8T, CD8Tex, Tprolif, and Treg cells at the single-cell level. (center) Illustrates the expression distribution of PSME3 in LIHC. (right) Presents a violin plot depicting the single-cell expression profile based on PSME3. [file Image_3.tif]
